# Pollen Viability and Anomalies in European Hazelnut: Cultivar Traits or Environmental Effect?

**DOI:** 10.3390/plants14233576

**Published:** 2025-11-23

**Authors:** Claudio Brandoli, Sonia Demasi, Valeria Fochi, Giovanni Caccialupi, Valerio Cristofori, Cristian Silvestri, Consolata Siniscalco, Claudio Todeschini, Elisabetta Sgarbi

**Affiliations:** 1 BIOGEST-SITEIA, University of Modena and Reggio Emilia, Via Amendola 2, 42124 Reggio Emilia, Italy; elisabetta.sgarbi@unimore.it; 2 Department of Life Science and Systems Biology, University of Turin, Viale Mattioli 25, 10125 Torino, Italy; sonia.demasi@unito.it (S.D.); valeria.fochi@unito.it (V.F.); consolata.siniscalco@unito.it (C.S.); 3 Department of Life Sciences, University of Modena and Reggio Emilia, Via Amendola 2, 42122 Reggio Emilia, Italy; giovanni.caccialupi@unimore.it; 4 Department of Agriculture and Forest Sciences (DAFNE), University of Tuscia, Via S. Camillo de Lellis snc, 01100 Viterbo, Italy; valerio75@unitus.it (V.C.); silvestri.c@unitus.it (C.S.); 5 Hazelnut Company Division, Ferrero Trading Lux S.A., Route de Trèves, L-2633 Senningerberg, Luxembourg; claudio.todeschini@ferrero.com

**Keywords:** *Corylus avellana* L., cultivar selection, pollen hydration, pollen sterility, pollinizer cultivars

## Abstract

Assessing pollen viability and anomalies is essential to optimize resources and improve hazelnut productivity. However, knowledge of pollen viability dynamics across cultivars and environments remains limited. This study applied impedance flow cytometry to (i) monitor pollen hydration and define optimal rehydration time, (ii) quantify pollen viability over three flowering seasons, and (iii) evaluate genetic, environmental, and agronomic influences on viable and anomalous pollen formation. Viable pollen showed an adaptive response, restoring high viability (~85%) after four hours of hydration following dehydration stress. Viability displayed cultivar-specific patterns, stable across years but variable among sites. In Viterbo (central Italy, Mediterranean climate), flowering occurred 2–4 weeks earlier than in northern orchards (Piedmont, continental climate). Wild-type accessions exhibited higher viability and minimal anomalous pollen (<3%), whereas cultivated genotypes maintained abundant anomalous pollen (30–50%) across sites and seasons. Multifactorial analysis revealed that both genotype and environment affected viable pollen, while anomalous pollen depended mainly on genotype. Overall, pollen viability results from the interaction between genetic predisposition and local conditions, whereas anomalous pollen reflects stable, genotype-linked traits. These findings highlight the dominant role of cultivar-specific genetics in hazelnut pollen quality, providing a framework for breeding and orchard management strategies.

## 1. Introduction

The European hazelnut (*Corylus avellana* L.) is an important global crop, ranking fifth in total nut production in recent years. Turkey is the leading producer in the global hazelnut market, accounting for approximately 75% of the total production, followed by Italy, the United States, Azerbaijan and the Republic of Georgia [1]. Hazelnuts also represent an important component of the sustainable economic growth of countries with productive potential such as Chile, due to their stable trade returns [2,3,4]. The current international outlook expects a further increase in demand for this product over the next decade [5], partly driven by growing consciousness of the health benefits associated with daily consumption of hazelnuts, owing to their content of unsaturated fatty acids, antioxidants, vitamins and proteins [6,7]. To meet this growing demand, producers must develop strategies to utilize their resources more efficiently. Consequently, studying the dynamics that govern the reproduction processes of this wind-pollinated perennial plant has become one of the main issues on the agenda of producing countries, particularly regarding the renewal of hazelnut orchards to enhance their competitiveness. Recent advancements in hazelnut cultivation result from the gradual evolution of the plant training systems [8] and planting layouts [9] which, however, has not been accompanied of the pool of cultivars used as pollinizers. Indeed, global production still relies on limited number of cultivars highly valued by the confectionery industry [10]. In hazelnut, a monoecious protogynous species, the receptivity of pistillate flowers extends for two to five weeks, depending on the cultivar [11]. During this extended period, successful pollination depends on several factors, including genetic compatibility, optimal weather conditions, nutrient availability, and the presence of viable pollen. Disruptions in any of these events can compromise fruit set and yield. It has been widely observed that pollen viability is strongly influenced by temperature as well as by cytoplasmic carbohydrate content. The presence of sucrose, the main pollen cytoplasmic osmolyte in pollen, can influence pollen rehydration, which is the first step following adhesion to a receptive and compatible stigma [12]. Hazelnut pollen, which is released with a relatively high moisture content (30%) compared to orthodox species (1–5% hydration) [13,14], is considered more susceptible to dehydration damage. Indeed, reduced moisture causes a rapid loss of viability [15], classifying hazelnut pollen as desiccation-sensitive [16]. Understanding flowering physiology is crucial for synchronizing the flowering times of production to pollinizer cultivars, especially in self-incompatible species such as hazelnut, where self-pollen can significantly limit fruit set. Furthermore, in hazelnut, key horticultural traits such as fruit weight and shape, peeling rate, and shell thickness are highly hereditable characteristics [17]. Pollen limitation, defined as a phenomenon in which pistils receive an insufficient number of pollen grains to fertilize ovules, has been shown to be an important factor in regulating fruit set [18]. Conversely, when the number of pollen grains available for pollination exceed the number of ovules, fertilization may not occur. This phenomenon is usually related to pollen population [19,20], referring to both the density and quality of pollen, including the presence of sterile or non-viable grains. Abnormal pollen development in angiosperms is commonly associated with alterations during meiosis or post-meiotic mitosis in the microspore [21]. These morphological changes have been largely attributed to genetic factors and environmental cues, with temperature playing a pivotal role [22]. Despite numerous studies documenting morphological anomalies in European hazelnut pollen [23,24,25,26,27], particularly in commercial genotypes, the relationship between pollen anomalies and genotypic traits, as well as the pedoclimatic conditions of the environment, remains largely unexplored. Anomalous pollen appears to be characterized by reproductive sterility associated with poor cytoplasm [28], callose accumulation, and a highly thickened intine [29]. Furthermore, anomalous pollen appears morphologically smaller than viable pollen, a conditions likely associated with recurrent reciprocal translocation events in *Corylus avellana* cultivars, leading to gametic semi-sterility [30,31].

While monitoring airborne pollen availability is a valid method for forecasting hazelnut annual yield [3], a more comprehensive understanding of pollen dispersal dynamics and pollen viability levels may help determine which cultivars are best suited to new growing regions, particularly in an era of increasingly irregular weather patterns resulting from global climate change. To expand current knowledge of hazelnut pollen characteristics and to support the development of artificial pollination technologies [32] and genetic improvement programs for this species [33], this study investigated pollen viability trends in four of the most appreciated Italian cultivars, along with a wild-type accession, monitored over three consecutive years. Particular attention was given to exploring the occurrence of anomalous pollen and its potential associations with genetic and environmental factors, thereby providing a clearer framework for understanding cultivar-specific pollen performance and its implications for cultivation strategies.

The proposed methodology and results are intended to facilitate future studies on hazelnut breeding, which often require simple, rapid, and reliable tests for assessing pollen quality. This study emerges as part of the project on the reproductive biology and pollen viability of the European hazelnut.

## 2. Results

### 2.1. Pollen Hydration Dynamics

**Figure 1:**
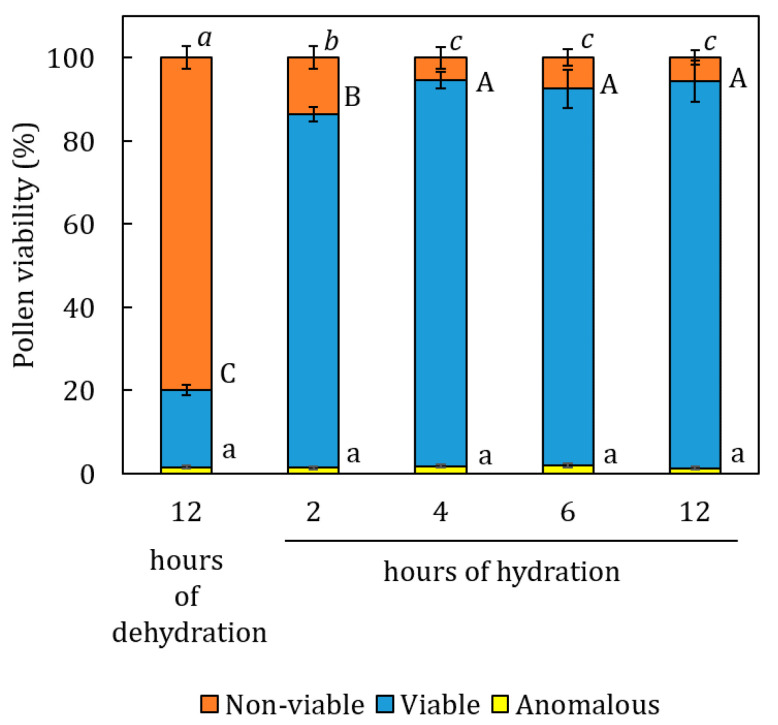
Trends in pollen viability after dehydration and subsequent rehydration test. Data are shown as means of at least three biological replicates. Error bars represent the standard error of the mean. Data for each pollen category (viable, anomalous and non-viable) were analyzed by Tukey’s test. Statistically differences (*p* < 0.05) are indicated by different letters (capital letters for viable pollen, italics for non-viable pollen, lowercase letters for anomalous pollen).

**Figure 2:**
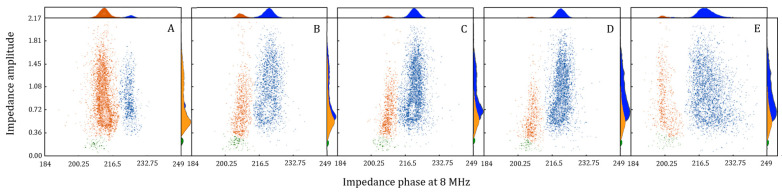
Effect of pollen hydration dynamics over time explored by IFC test. Pollen after (**A**) 12 h of dehydration and subsequent rehydration of (**B**) 2 h, (**C**) 4 h, (**D**) 6 h and (**E**) 12 h. Blue dots correspond to viable pollen, red dots correspond to non-viable pollen and green dots to anomalous pollen. Data are shown as means of at least three biological replicates.

Pollen hydration dynamics tests showed, following the preparatory dehydration phase, a pronounced presence of non-viable pollen (80%) compared to viable (18.5%) and anomalous pollen (1.5%). Viability levels increased rapidly from 18.6% to 84.9% within two hours of hydration, reaching a plateau after four hours (Figure 1). During this hydration phase, the percentage of pollen initially classified as non-viable decreased rapidly, from 79.9% to 13.6% after only two hours, ultimately stabilizing at approximately 7% after four hours. The presence of anomalous pollen remained constant throughout the experiment, consistently below 3%. IFC analysis of pollen hydration dynamic highlighted that the separation between impedance phase values of viable and non-viable pollen increased with hydration time (Figure 2).

### 2.2. Pollen Viability

**Figure 3:**
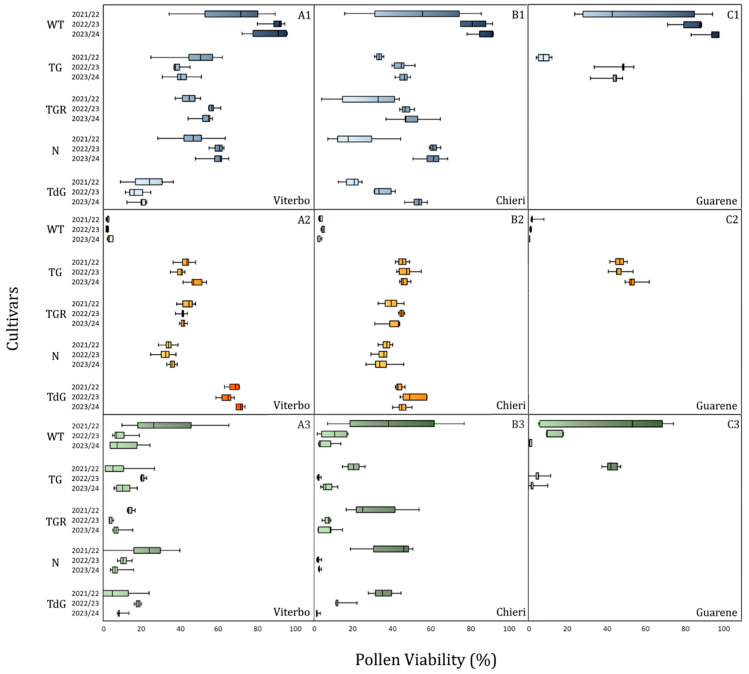
Percentage of viable (**A1**–**C1**), anomalous (**A2**–**C2**) and non-viable (**A3**–**C3**) pollen of the cultivars Tonda Gentile (TG), Tonda Gentile Romana (TGR), Nocchione (N), Tonda di Giffoni (TdG) and of a wild type (WT) accession of the varietal fields of Viterbo (**A1**–**A3**), Chieri (**B1**–**B3**) and Guarene (**C1**–**C3**) during the 2021/22, 2022/23 and 2023/24 flowering seasons. Data are shown as means of at least three biological replicates.

**Figure 4:**
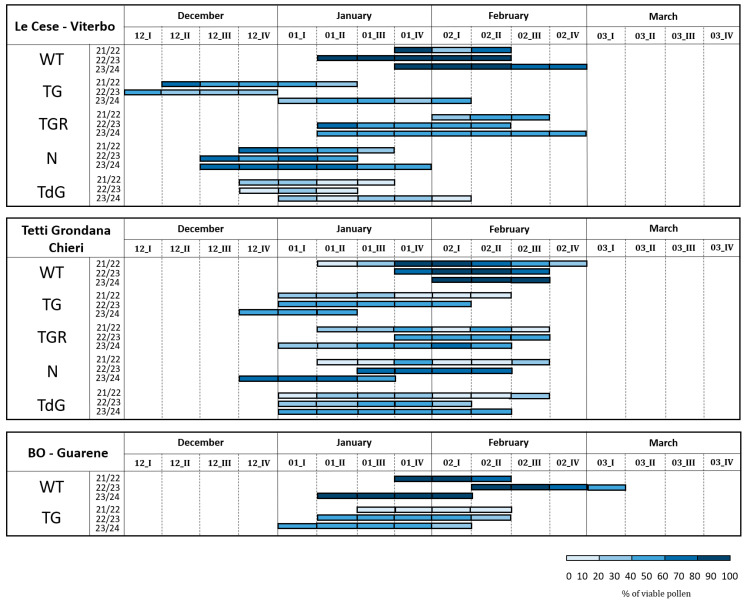
Flowering time of the cultivars Tonda Gentile (TG) syn. ‘Tonda Gentile delle Langhe’, Tonda Gentile Romana (TGR), Nocchione (N) and Tonda di Giffoni (TdG) in association with a wild type (WT) accession during the 2021/22, 2022/23, 2023/24 flowering seasons. The phenogram represents both the flowering time and the percentage of pollen viability per week with light blue bars of increasing intensity. “Beginning” indicates the time when few catkins start to release pollen. “Full flowering” indicates the time when the peak of dispersal is reached (about 50% of catkins release pollen), and “end” indicates the last part of flowering when few catkins still release pollen.

The results of the IFC analysis of pollen viability, measured after four hours of hydration, are shown in Figure 3. The percentage of viable pollen released by each genotype over the three-year period exhibited a consistent pattern. This was particularly evident in the ‘Le Cese’ field (Viterbo province), where each genotype displayed a distinct and narrow range of values. In this field, the cultivars TG, TGR and N consistently exhibited intermediate viability levels between 20 and 60%, whereas TdG was always characterized by lower percentages, ranging from 10 to 30% (Figure 3(A1)). This similarity of mean values among seasons was accompanied by a highly variable period of pollen dispersal, especially in the Viterbo area, with a shift in floral phenology ranging from two to four weeks between seasons, mostly for the cultivars TG, TGR, and the WT accession (Figure 4). Contrary to observations in Viterbo, the northern areas of Italy, namely the Chieri and Guarene fields, exhibited greater fluctuations in viability levels during the three flowering years, while still maintaining some consistency (Figure 3(B1)). Similar to observations in Viterbo, the TG, TGR, and N cultivars in the Chieri area showed intermediate levels of viability, between 30 and 65%, except for the first sampling year which showed lower percentages. Even in the Guarene orchard, TG values were comparable to those observed for the same genotype in the other two fields, though exhibiting greater variability in the first sampling year compared to the other two (Figure 3(C1)). The flowering time in the northern orchards followed a more consistent pattern, characterized by high homogeneity among the seasons. All cultivars release pollen between the last week of December and the first half of February, with the only exceptions being TGR and N, which showed a flowering delay of 2–3 weeks (Figure 4).

Overall, WT accessions from all varietal fields exhibited values substantially higher than the remaining cultivars. The only exception was the first year of observation, when values in all areas showed greater fluctuations (Figure 3(A1–C1))

**Figure 5:**
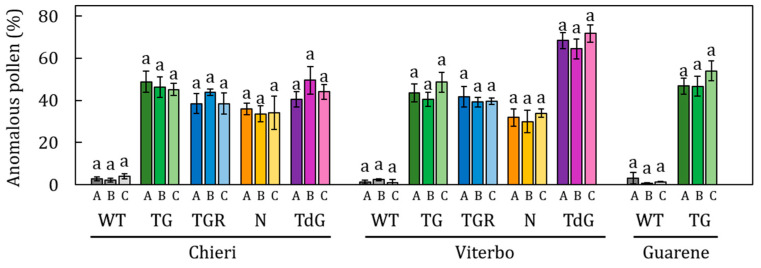
Comparison of the percentage of total anomalous pollen released during the flowering seasons 2021–22 (A), 2022–23 (B) and 2023–24 (C) belonging to the cultivars Tonda Gentile Romana (TGR), Nocchione (N), Tonda Gentile (TG), Tonda di Giffoni (TdG) and a Wild type (WT) accession in the varietal fields of Chieri, Viterbo and Guarene. Data are shown as means of at least three biological replicates. Error bars represent the standard error of the mean. Data collected in each flowering season for each cultivar in each single varietal field were analyzed using Tukey’s test. Statistically differences (*p* < 0.05) are indicated with different letters.

The total concentration of anomalous pollen detected in WT accessions never exceeded 3%, whereas cultivated genotypes exhibited stable and abundant levels across all three flowering seasons, oscillating between 30 and 50%, depending on the cultivar (Figure 3(A2–C2)). Comparing the total values of anomalous pollen released within each cultivar during the three-year period, no significant differences were observed between seasons (Figure 5).

**Figure 6:**
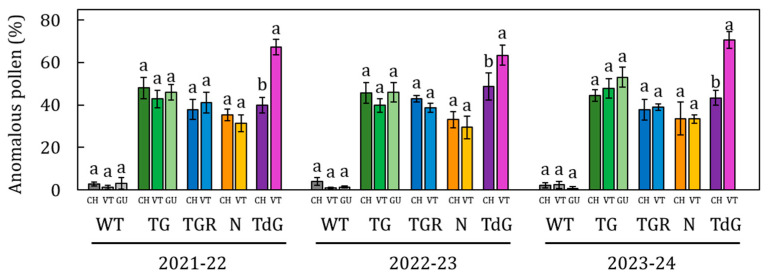
Comparison of the percentage of total anomalous pollen shed among the fields of Chieri (CH), Viterbo (VT) and Guarene (GU) of the cultivars Tonda Gentile (TG), Tonda Gentile Romana (TGR) Nocchione (N), Tonda di Giffoni (TdG) and a Wild type (WT) accession during the flowering seasons 2021–22, 2022–23, 2023–24. Data are shown as means of at least three biological replicates. Error bars represent the standard error of the mean. Data from different collection fields for each cultivar in each single flowering season were analyzed using Tukey’s test. Statistically differences (*p* < 0.05) are indicated with different letters.

Similarly, the comparison of the anomalous pollen values across the three varietal fields for each cultivar did not reveal significant differences. The only exception was TdG in Viterbo, which stood out with higher average values (68.16%) than TdG in Chieri (Figure 6). Overall, the average percentage of anomalous pollen dispersed in each field was homogeneous, with values of 46% in Viterbo, 41% in Chieri, and 49% in Guarene, with no significant differences among the fields. The content of non-viable pollen negatively reflected that of the viable one. This is particularly evident in the ‘Le Cese’, where the fluctuations were lower than in the remaining fields (Figure 3(A3–C3)).

To verify potential correlations between genetic and environmental factors in the formation of viable and anomalous pollen, a multifactorial ANOVA was conducted on cultivars of commercial interest. The results showed that both viable and anomalous pollen were strongly influenced by the geographical area (representing the environmental factor) and the genotype. The relevance (R^2^) of the ‘genotype’ factor was consistently higher than that of the environmental factor for both parameters, especially for anomalous pollen (Table 1). The phenological phase of collection, however, had a significant effect only during the 2021–22 and 2022–23 flowering seasons on viable pollen, while it had no effect on the anomalous one. All interactions between variables, including the multi-factor interaction F × G × PP of collection, significantly influenced (*p* ≤ 0.001) the state of viable pollen, while the ‘environmental’ factor and ‘genotype’ consistently had a significant effect on the formation of anomalous pollen (Table 1).

Two-way ANOVA conducted on WT accessions showed a significant effect (*p* value ≤ 0.001) of the factors ‘environment’ and ‘phenological phase of collection’ on pollen viability throughout the three-year period. The relevance (R^2^) of the phenological phase increased over the years, from 1.7% to 38.5%, while the factor ‘environment’ remained stable, on average lower than in cultivars. All interactions between variables significantly affected the formation of viable pollen (Table 2). Similarly to what was observed in commercial genotypes, analysis of anomalous pollen content revealed that the time of collection did not alter its concentration, and the interaction between environment × phenological phase was never significant. Furthermore, it was found that the area of origin has little relevance for anomalous pollen formation; in particular, during the first year of monitoring this factor did not show any significance (Table 2).

## 3. Discussion

The analysis of pollen hydration dynamics conducted in WT pollen highlighted the importance of hydration in maintaining pollen viability during dispersal. Indeed, in wind-pollinated species, pollen viability and phenology were found to be largely influenced by climatic conditions during the flowering period [26,34,35]. This correlation, strongly dependent on the moisture content of pollen at dispersal, is particularly evident in species known to be sensitive to dehydration, such as hazelnut [16]. This condition makes it more susceptible to dehydration damage [15]. The separation of IFC phase values observed between viable and non-viable pollen (Figure 2), which is directly proportional to the increase in hydration time, suggests easier detection of pollen impedance values. This phenomenon is related to the re-acquired structural conformation of the external membrane [36] and to the re-activation of metabolic activity [29]. Furthermore, the recovered levels of pollen viability after an adequate hydration phase support the previously proposed hypothesis of an acquired adaptive strategy of hazelnut pollen to overcome adverse climatic conditions during winter flowering [28]. Regarding anomalous pollen, always detected at low and constant levels in the WT accessions during the entire dehydration/rehydration process, this observation suggests the absence of correlation with metabolic alteration and cell damage. The anomaly, characterized by reproductive sterility, would be linked to scanty cytoplasm, accumulation of callose and very thickened intine [37]. Furthermore, the group of anomalous pollen detected at IFC was always characterized by lower average amplitudes, indicative of smaller average dimensions (Figure 2), as suggested by some authors [23,25,26]. The genetic and non-environmental nature of this category would be further supported by the results obtained in this study.

The analysis of pollen viability revealed largely similar trends within each cultivar over three years of observation, suggesting a cultivar-specific trait (Figure 3(A1–C1)). This hypothesis is confirmed by the analysis of variance, showing that the ‘genotype’ factor played a pivotal role (30 ≤ R^2^ ≤ 45%) in determining the presence of viable pollen throughout the entire period (Table 1). Similarly, the variability detected among seasons and collection fields may reflect exposure to different environmental conditions, as suggested in the literature [38,39]. Hazelnut is a winter-flowering plant, releasing pollen over an extended period. Climatic fluctuations occurring during this critical phase can influence pollen susceptibility [40]. The seasonal trend of pollen viability is therefore the result of a precise tuning between intrinsic genetic traits of each cultivar and the pedoclimatic conditions of the growing sites. The ‘genotype’ effect would therefore predispose to certain levels of viability, while ‘environmental factors’, including soil properties, exposure, and orchard management practices, modulate these levels to a limited extent.

Although all interaction terms were statistically significant (*p* < 0.001), data examination suggests that most variability arises from phenological differences among cultivars rather than from year-to-year changes. Specifically, while pollen viability percentages remain relatively stable across seasons and sites for each genotype (Figure 3), flowering onset varies considerably across locations, with flowering occurring 2–4 weeks earlier in Viterbo than in northern sites (Figure 4). These results indicate that E × G and E × PP interactions are biologically significant, mainly reflecting cultivar-specific phenological responses to local climatic conditions, with small contributions from interannual variability in pollen viability.

The limited variability observed in the proportion of anomalous pollen grains within each cultivar further sugests that ‘phenological/climatic’ factors have negligible influence on the morpho-physiological development of this pollen type. Instead, their occurrence appears to be predominantly determined by genetic factors. Linear correlation analysis supported this conclusion, showing that the incidence of anomalous pollen was largely associated with the genotype (35 ≤ R^2^ ≤ 52%) rather than environmental conditions (1.8 ≤ R^2^ ≤ 4.1%), attributing the origin and formation of this pollen to irregularities occurring during meiosis of the mother cells [30,31]. It has been demonstrated that crossing-over during meiosis between homologous chromatids can cause significant genomic deletions, with consequent effects on cellular metabolic balance [41]. Anomalous pollen grains would therefore be characterized by little or total metabolic inactivity due to the loss of a considerable portion of the genome, as reported in *Zea mays* L. [42] and *Typha latifolia* [43].

Overall, our results indicate that European hazelnut pollen is highly sensitive to hydration, as reflected in variations of impedance signals. Pollen viability thus depends on an optimal hydration status, which allows the expression of cultivar-specific genetic potential. Within this framework, genetic factors serve as predisposing determinants of viability levels, whereas local pedoclimatic conditions and orchard management practices modulate the physiological processes that maintain pollen hydration throughout the season. Finally, a high percentage of anomalous pollen was observed in the main Italian genotypes of commercial interest. This characteristic was found to be strongly dependent on intrinsic genetic factors rather than specific climatic conditions. The obtained results are expected to have not only ecophysiological inferences, but also give practical guidelines for future breeding programs [33] and for the development of finalized artificial pollination technologies [32].

## 4. Materials and Methods

### 4.1. Plant Materials and Study Areas

In this study, four of the most internationally appreciated Italian hazelnut cultivars, Tonda Gentile’ sin. ‘Tonda Gentile delle Langhe’ (TG), ‘Tonda Gentile Romana’ (TGR), ‘Nocchione’ (N) and ‘Tonda di Giffoni’ (TdG) were considered, in association with a late-flowering wild type (WT) accession, identified for each experimental site. Each cultivar was represented by a pool of 10–15 genetically certified plants. The samples were selected from three varietal fields located in two different regions of northern and central Italy (Table 3).

The 12-year-old plants in Chieri and Guarene and the 24-year-old plants in Viterbo were grown according to the multi-stem bush model and planted at a distance of 5 × 4 m. In Chieri varietal field, no mineral fertilization was carried out and no irrigation was performed. In Guarene, orchard irrigation was applied with 20 mm every 15 days from mid-June to the end of July; 500 kg ha^−1^ of NPK (12:12:17) fertilizer, with the addition in autumn of mature manure, were applied every two years. In Viterbo varietal field, namely ‘Le Cese’, the orchard was irrigated by a subirrigation system and managed with standard management techniques, receiving annual applications of nitrogen 90 kg ha^−1^, phosphorus 60 kg ha^−1^ and potassium 90 kg ha^−1^.

The pedological characteristics of the two northern orchards of Chieri and Guarene are quite similar. Both areas are characterized by a silty-clayey soil with poor skeleton and a sub-alkaline reaction and medium exchange capacity. According to Regione Piemonte Soil Map, the content of organic matter for Chieri is 1.28% and for Guarene is 1.15% [44]. Differently, the third varietal field is located near the crater lake of Vico, which originated following the filling of the polygenic caldera of the volcano with the same name. The surrounding area is characterized by a sandy-clayey texture with debris from the aquifer, sands and reworked tuffs [45]. The territory is characterized by an acid/sub-acid pH [46] and a very compact skeleton, consisting of a dense stratification of lapilli, humidity and a partial constitution of ignimbrite, rich in lava pebbles and content of organic matter of 1.1% [47,48,49,50].

The two study sites are characterized by distinct climates, reflecting the environmental differences of each region. According to the Köppen–Geiger classification, Chieri falls within the Cfa (humid temperate) zone, while Viterbo is classified as Csa (hot summer Mediterranean). At the Chieri site, winters are cold and humid, with minimum temperatures frequently falling below 0 °C. Spring and autumn are generally mild, and summers are hot and humid, with average maximum temperatures often exceeding 30 °C in July and August (https://servizi.regione.piemonte.it/ , accessed on 30 October 2025). In contrast, Viterbo experiences milder winters, with average minimum temperatures around 3–4 °C. Precipitation is mainly concentrated in autumn and, to a lesser extent, in spring. Summers are hot and dry, with average daily maximum temperatures exceeding 30 °C from June to August [51].

### 4.2. Pollen Collection and Conservation

Pollen from each cultivar was collected weekly for three consecutive years, during the 2021/22, 2022/23 and 2023/24 flowering seasons, from December to March. During this period, plants were monitored to determine the phenological phases of each cultivar, following the standardized BBCH scale for hazelnuts [52]. Five/six inflorescences per plant were collected exclusively from fully elongated catkins, in different parts of the canopy, according to the different cardinal exposures and selected from at least three different shrubs per cultivar. Once picked up, catkins were left to dry overnight at room temperature to facilitate pollen release. The pollen was then collected and mixed in Eppendorf tubes, according to the field, genotype and collection date. Finally, it was stored at low temperature (+4 °C) to be analyzed in the following days, according to Brandoli et al. [26].

### 4.3. Study on Pollen Hydration Dynamics

It has been widely reported that pollen viability is strongly influenced by its hydration state [53,54,55]. In particular, hazelnut pollen is released with a relatively high moisture content (30%), which makes it more susceptible to dehydration damage during the winter dispersal period, resulting in a rapid loss of viability [26]. For this reason, it was essential to establish the best pollen hydration time before proceeding with the analyses. A preparatory test was performed using a WT accession. It is known that these accessions in hazelnut are characterized by high levels of viability, higher than commercial cultivars [25,56]. Furthermore, WT accessions are characterized by low levels of anomalous pollen. These characteristics together ensured a simpler interpretation of the results by limiting the classification of pollen into viable and non-viable.

WT pollen, collected during the 2021–2022 flowering season, was mixed in a single eppendorf and subsequently dehydrated overnight (12 h) on silica gel and the viability levels immediately analyzed. Pollen viability was subsequently estimated after 2, 4, 6 and 12 h of rehydration in a humidity chamber. For each dehydration-hydration step, viability determination was performed in triplicate.

### 4.4. Pollen Viability Analysis

Pollen viability was assessed by using the impedance flow cytometry (IFC) Ampha^TM^ Z32 instrument, software version 2.1.6 (Amphasys, Technopark Lucerne, Root, Switzerland), which represents the new frontier for a fast and label-free analysis of pollen grains in various plant species. This technology, evolved from the studies of Cheung et al. [57], is able to provide information on the state of viability by exploiting the dielectric properties of pollen. When each individual pollen grain passes through the electric field at specific frequencies, it changes the detected impedance signal. This happens since the polarization of the cell membranes decreases and a variation of the capacitance and conductance of the pollen can be detected and measured [58]. Before analysis, pollen samples were dry-sieved with a 50 µm filter to remove any plant debris. After rehydration, pollen was suspended in 1 mL of isosmotic IFC buffer for 5 min (AF6 buffer—Amphasys, Root, Switzerland) to facilitate the separation of the grains, and further filtered and re-diluted with 1 mL of AF6 buffer in FACS tubes, to avoid any clogging of the chip. Pollen was then pumped into a 120 μm × 120 μm microchannel chip to which an electric field was applied at intermediate frequencies (1–8 MHz). The flow of analysis was 1000/s pollen grains for approximatively 130,000 pollen grains per sample analyzed. The analysis classifies the data into viable, non-viable and anomalous pollen. The data obtained were then normalized to 100 after eliminating all those for which it was not possible to determine a specific category, such as plant debris, air bubbles and dust. Viability determination was always performed in triplicate.

### 4.5. Data Analysis Procedure

Statistical differences between accessions were assessed using Student’s t-test, ANOVA and Tukey post hoc test (*p* ≤ 0.05). A multivariate regression approach using analysis of variance (ANOVA) was performed for the dataset belonging to commercial genotypes only for each flowering season to describe the significance (*p* ≤ 0.001) between environment, genotype and phenological stage of harvest on the presence of viable and anomalous pollen. For wild type accessions, a two-way ANOVA test was performed to better recognize the significance (*p* ≤ 0.001) of environment and phenological phase of collection. In both case Pearson’s linearity test was applied to understand the performance of each single parameter according to Tai [59].

ANOVA analyses were performed using GENSTAT 17th edition software (VSN International, Hemel Hempstead, UK).

## Figures and Tables

**Table 1:** Significance by multifactorial ANOVA test on commercially interesting genotypes of the environment (E), genotype (G) and phenological phase of collection (PP), as well as of the interactions environment × genotype (E × G), environment × phenological phase of collection (E × PP), genotype × phenological phase of collection (G × PP) and environment × genotype × phenological phase of collection (E × G × PP) on viable and anomalous pollen formation (* = *p* ≤ 0.001). Data are reported by flowering season (2021–22, 2022–23, 2023–24). n.s. means not significant.

	Environment (E)	R^2^ (E) %	Genotype (G)	R^2^ (G) %	Phenological Phase (PP)	R^2^ (PP) %	E × G	E × PP	G × PP	E × G × PP
*Season 2021–22*										
Viable pollen	*	10.4	*	32.7	*	7.2	*	*	*	*
Anomalous pollen	*	1.8	*	38.3	n.s.	n.s.	*	n.s.	n.s.	n.s.
*Season 2022–23*										
Viable pollen	*	25.1	*	35.1	*	3.4	*	*	*	*
Anomalous pollen	*	3.7	*	51.8	n.s.	n.s.	*	n.s.	n.s.	n.s.
*Season 2023–24*										
Viable pollen	*	19.9	*	44.1	n.s.	n.s.	*	*	*	*
Anomalous pollen	*	4.1	*	36.4	n.s.	n.s.	*	n.s.	n.s.	n.s.

**Table 2:** Significance for wild type accessions by two-way ANOVA of environment (E) and phenological phase of collection (PP), as well as the interaction environment × phenological phase of collection (E × PP) on viable and anomalous pollen (* = *p* ≤ 0.001). Data are reported by flowering season (2021–22, 2022–23, 2023–24). n.s. means not significant.

	Environment (E)	R^2^ (E) %	Phenological Phase (PP)	R^2^ (PP) %	E × PP
*Season 2021–22*					
Viable pollen	*	3.6	*	1.7	*
Anomalous pollen	n.s.	n.s.	n.s.	n.s.	n.s.
*Season 2022–23*					
Viable pollen	*	4.7	*	16.3	*
Anomalous pollen	*	6.5	n.s.	n.s.	n.s.
*Season 2023–24*					
Viable pollen	*	7.7	*	38.5	*
Anomalous pollen	*	0.54	n.s.	n.s.	n.s.

**Table 3:** Area and geographical coordinates of the varietal fields.

Area	Field Name	Cultivars Analyzed	Coordinates
Chieri, Piedmont	Tetti Grondana	WT, TG, TGR, N, TdG	Lat. 45°02′29″ N, long. 7°50′08″ E, AMSL 327 m
Guarene, Piedmont	BO	WT, TG	Lat. 44°44′08″ N, long. 8°02′25″ E, AMSL 167 m
Caprarola, Lazio	Le Cese	WT, TG, TGR, N, TdG	Lat. 42°20′00″ N; long. 12°11′00″ E; AMSL 570 m

## Data Availability

The original contributions presented in this study are included in the article. Further inquiries can be directed to the corresponding author

## References

[1] FAO Food and Agriculture Organization of the United Nations. https://www.fao.org/home/en.

[2] Ellena M., Sandoval P., Gonzalez A., Galdames R., Jequier J., Contreras M., Azocar G. (2014). PRELIMINARY RESULTS OF SUPPLEMENTARY POLLINATION ON HAZELNUT IN SOUTH CHILE. Acta Hortic..

[3] Ascari L., Siniscalco C., Palestini G., Lisperguer M.J., Suarez Huerta E., De Gregorio T., Bregaglio S. (2020). Relationships between Yield and Pollen Concentrations in Chilean Hazelnut Orchards. Eur. J. Agron..

[4] Mohr Fuchslocher J.V., Navarro Gaete S.A. (2023). Hazelnut Production Areas in Chile, Performance of Cultivars from Oregon State University, and an Equation to Predict Performance. Acta Hortic..

[5] WTO World Trade Organization. https://www.wto.org/search.

[6] Köksal A.İ., Artik N., Şimşek A., Güneş N. (2006). Nutrient Composition of Hazelnut (*Corylus avellana* L.) Varieties Cultivated in Turkey. Food Chem..

[7] Wani I.A., Ayoub A., Bhat N.A., Dar A.H., Gull A., Nayik G.A., Gull A. (2020). Hazelnut. Antioxidants in Vegetables and Nuts-Properties and Health Benefits.

[8] Pacchiarelli A., Silvestri C., Muganu M., Cristofori V. (2025). Influence of the Plant Training System on Yield and Nut Traits of European Hazelnut (*Corylus avellana* L.) Cultivar Nocchione. Agronomy.

[9] Balta F., Yılmaz M., Karakaya O., Çalışkan K., Yarılgaç T., Bostan S.Z., Balta M.F., Uzun S. (2024). Effect of Plant Density on Nut Traits, Nut Yield, Cluster Distribution and Chemical Components in Çakıldak (*Corylus avellana* L.) Hazelnut Cultivar. Appl. Fruit Sci..

[10] Mehlenbacher S.A. (2009). GENETIC RESOURCES FOR HAZELNUT: STATE OF THE ART AND FUTURE PERSPECTIVES. Acta Hortic..

[11] Pacchiarelli A., Lupo M., Ferrucci A., Giovanelli F., Priori S., Pica A.L., Silvestri C., Cristofori V. (2024). Phenology, Yield and Nut Traits Evaluation of Twelve European Hazelnut Cultivars Grown in Central Italy. Forests.

[12] Heslop-Harrison J. (1979). AN INTERPRETATION OF THE HYDRODYNAMICS OF POLLEN. Am. J. Bot..

[13] Nepi M., Franchi G.G., Padni E. (2001). Pollen Hydration Status at Dispersal: Cytophysiological Features and Strategies. Protoplasma.

[14] Pacini E., Guarnieri M., Nepi M. (2006). Pollen Carbohydrates and Water Content during Development, Presentation, and Dispersal: A Short Review. Protoplasma.

[15] Zielinski Q.B. (1968). Techniques for Collecting, Handling Germinating, and Storing of Pollen of the Filbert (*Corylus* Spp.). Euphytica.

[16] Franchi G.G., Piotto B., Nepi M., Baskin C.C., Baskin J.M., Pacini E. (2011). Pollen and Seed Desiccation Tolerance in Relation to Degree of Developmental Arrest, Dispersal, and Survival. J. Exp. Bot..

[17] Fattahi R., Mohammadzedeh M., Khadivi-Khub A. (2014). Influence of Different Pollen Sources on Nut and Kernel Characteristics of Hazelnut. Sci. Hortic..

[18] Ashman T.-L., Knight T.M., Steets J.A., Amarasekare P., Burd M., Campbell D.R., Dudash M.R., Johnston M.O., Mazer S.J., Mitchell R.J. (2004). POLLEN LIMITATION OF PLANT REPRODUCTION: ECOLOGICAL AND EVOLUTIONARY CAUSES AND CONSEQUENCES. Ecology.

[19] Brewbaker J.L., Majumder S.K. (1961). CULTURAL STUDIES OF THE POLLEN POPULATION EFFECT AND THE SELF-INCOMPATIBILITY INHIBITION. Am. J. Bot..

[20] Hormaza J.I., Herrero M. (1996). Dynamics of Pollen Tube Growth under Different Competition Regimes. Sex. Plant Reprod..

[21] Larrosa F.H., Maune J.F., Erazzú L.E., Camadro E.L. (2012). Meiotic Abnormalities Underlying Pollen Sterility in Wild Potato Hybrids and Spontaneous Populations. Plant Biol..

[22] Iovane M., Aronne G. (2022). High Temperatures during Microsporogenesis Fatally Shorten Pollen Lifespan. Plant Reprod..

[23] Frenguelli G., Ferranti F., Tedeschini E., Andreutti R. (1997). Volume Changes in the Pollen Grain of *Corylus avellana* L. (Corylaceae) during Development. Grana.

[24] Novara C., Ascari L., La Morgia V., Reale L., Genre A., Siniscalco C. (2017). Viability and Germinability in Long Term Storage of *Corylus avellana* Pollen. Sci. Hortic..

[25] Ascari L., Cristofori V., Macrì F., Botta R., Silvestri C., De Gregorio T., Huerta E.S., Di Berardino M., Kaufmann S., Siniscalco C. (2020). Hazelnut Pollen Phenotyping Using Label-Free Impedance Flow Cytometry. Front. Plant Sci..

[26] Brandoli C., Cristofori V., Silvestri C., Todeschini C., Sgarbi E. (2024). The Development of an Improved Medium for the In Vitro Germination of *Corylus avellana* L. Pollen. Forests.

[27] Brandoli C., Dito G., Tombesi S., Todeschini C., Siniscalco C., Sgarbi E. (2025). Relationship among Carbohydrates Content, Viability and Germinability in Pollen of European Hazelnut (*Corylus avellana* L.) Cultivars. Hortic. Environ. Biotechnol..

[28] Brandoli C., Mortada A., Todeschini C., Siniscalco C., Sgarbi E. (2024). The Role of Sucrose in Maintaining Pollen Viability and Germinability in *Corylus avellana* L.: A Possible Strategy to Cope with Climate Variability. Protoplasma.

[29] Edlund A.F. (2004). Pollen and Stigma Structure and Function: The Role of Diversity in Pollination. Plant Cell Online.

[30] Salesses G. (1973). Cytological Study of Genus Corylus: A Heterozygotic Translocation in Some Low Male Fertile Varieties of Hazelnut (*Corylus avellana*). Ann. Amelior. Plantes.

[31] Salesses G., Bonnet A. (1988). Cytogenetic Studies of Hybrides among *Corylus avellana* Having Translocations in Heterozygotic States. Cytologia.

[32] Broussard M.A., Coates M., Martinsen P. (2023). Artificial Pollination Technologies: A Review. Agronomy.

[33] Ferrucci A., Lupo M., Turco S., Pavese V., Marinoni D.T., Botta R., Cristofori V., Mazzaglia A., Silvestri C. (2023). A Roadmap of Tissue Culture and Biotechnology in European Hazelnut (*Corylus avellana* L.). Plant Physiol. Biochem..

[34] Črepinšek Z., Štampar F., Kajfež-Bogataj L., Solar A. (2012). The Response of *Corylus avellana* L. Phenology to Rising Temperature in North-Eastern Slovenia. Int. J. Biometeorol..

[35] Capik J.M., Molnar T.J. (2014). Flowering Phenology of Eastern Filbert Blight-Resistant Hazelnut Accessions in New Jersey. HortTechnology.

[36] Shivanna K.R., Heslop-Harrison J. (1981). Membrane State and Pollen Viability. Ann. Bot..

[37] Chicchiriccò G. (1989). Microsporogenesis and Pollen Development in *Crocus sativus* L. *Caryologia* **1989**, *42*, 249–257. Caryologia.

[38] Issarakraisila M. (1994). Effects of Temperature on Pollen Viability in Mango Cv. “Kensington”. Ann. Bot..

[39] Pacini E., Dolferus R. (2019). Pollen Developmental Arrest: Maintaining Pollen Fertility in a World With a Changing Climate. Front. Plant Sci..

[40] Jha P.K., Materia S., Zizzi G., Costa-Saura J.M., Trabucco A., Evans J., Bregaglio S. (2021). Climate Change Impacts on Phenology and Yield of Hazelnut in Australia. Agric. Syst..

[41] Lysák M.A., Schubert I., Greilhuber J., Dolezel J., Wendel J.F. (2013). Mechanisms of Chromosome Rearrangements. Plant Genome Diversity Volume 2.

[42] Brock R.D., Pryor A.J. (1996). An Unstable Minichromosome Generates Variegated Oil Yellow Maize Seedlings. Chromosoma.

[43] Berdnikov V.A., Kosterin O.E., Bogdanova V.S. (2002). Mortality of Pollen Grains May Result from Errors of Meiosis: Study of Pollen Tetrads in Typha Latifolia L. *Heredity* **2002**, *89*, 358–362. Heredity.

[44] Geoportale Regione Piemonte Home—Geoportale Piemonte. https://geoportale.igr.piemonte.it/cms/.

[45] ArpaLazio Piano Della Caratterizzazione Del Lago Di Vico 2012. https://www.arpalazio.it/documents/20124/40140/PdC_Vico_Regione.pdf.

[46] Dell’Abate M.T., Benedetti A., Nardi P., Di Bartolomeo E., Fabrizio G. (2009). Soil-Plant Relationships in the Cimini-Sabatini Hazelnut District: Plant Nutrition and Soil Fertility Status. Acta Hortic..

[47] Locardi E. (1965). Tipi Di Ignimbriti Di Magmi Mediterranei: Le Ignimbriti Del Vulcano Di Vico. Atti Soc. Toscana Sci. Nat..

[48] Barbanti L. (1969). Lago Di Vico: Rilevamento Batimetrico e Note Geomorfologiche. Mem. Dellistituto Ital. Idrobiol..

[49] Bertagnini A., Sbrana A. (1986). Il Vulcano Di Vico: Stratigrafia Del Complesso Vulcanico e Sequenze Eruttive Delle Formazioni Piroclastiche. Mem. Soc. Geol. Ital..

[50] Global Soil Organic Carbon Map Global Soil Organic Carbon Map (GSOCmap)|FAO SOILS PORTAL|Food and Agriculture Organization of the United Nations. https://www.fao.org/soils-portal/data-hub/soil-maps-and-databases/global-soil-organic-carbon-map-gsocmap/en/.

[51] ArpaLazio Inquadramento Climatologico Regione Lazio 2019. https://progetti.regione.lazio.it/contrattidifiume/app/uploads/sites/53/C_03_inquadramento-climatologico.pdf.

[52] Taghavi T., Rahemi A., Suarez E. (2022). Development of a Uniform Phenology Scale (BBCH) in Hazelnuts. Sci. Hortic..

[53] Hoekstra F.A., Crowe J.H., Crowe L.M. (1991). Effect of Sucrose on Phase Behavior of Membranes in Intact Pollen of *Typha Latifolia* L., as Measured with Fourier Transform Infrared Spectroscopy. Plant Physiol..

[54] Speranza A., Calzoni G.L., Pacini E. (1997). Occurrence of Mono- or Disaccharides and Polysaccharide Reserves in Mature Pollen Grains. Sex. Plant Reprod..

[55] Pacini E., Hesse M. (2004). Cytophysiology of Pollen Presentation and Dispersal. Flora-Morphol. Distrib. Funct. Ecol. Plants.

[56] Brandoli C., Sgarbi E., Cristofori V., Todeschini C., Siniscalco C. (2025). Correlation between Carbohydrate Content and Viability in Pollen of Some Italian Hazelnut Cultivars. Acta Hortic..

[57] Cheung K., Gawad S., Renaud P. (2005). Impedance Spectroscopy Flow Cytometry: On-Chip Label-Free Cell Differentiation. Cytometry A.

[58] Sun T., Morgan H. (2010). Single-Cell Microfluidic Impedance Cytometry: A Review. Microfluid. Nanofluidics.

[59] Tai G.C.C. (1979). Analysis of Genotype-Environment Interactions of Potato Yield^1^. Crop Sci..

